# Climate Envelope Modeling and Dispersal Simulations Show Little Risk of Range Extension of the Shipworm, *Teredo navalis* (L.), in the Baltic Sea

**DOI:** 10.1371/journal.pone.0119217

**Published:** 2015-03-13

**Authors:** Christin Appelqvist, Zyad K. Al-Hamdani, Per R. Jonsson, Jon N. Havenhand

**Affiliations:** 1 Department of Biological & Environmental Sciences, University of Gothenburg, Tjärnö, 452 96 Strömstad, Sweden; 2 Geological Survey of Denmark and Greenland, Øster Voldgade 10, DK-1350 Copenhagen K, Denmark; Technical University of Denmark, DENMARK

## Abstract

The shipworm, *Teredo navalis*, is absent from most of the Baltic Sea. In the last 20 years, increased frequency of *T*. *navalis* has been reported along the southern Baltic Sea coasts of Denmark, Germany, and Sweden, indicating possible range-extensions into previously unoccupied areas. We evaluated the effects of historical and projected near-future changes in salinity, temperature, and oxygen on the risk of spread of *T*. *navalis* in the Baltic. Specifically, we developed a simple, GIS-based, mechanistic climate envelope model to predict the spatial distribution of favourable conditions for adult reproduction and larval metamorphosis of *T*. *navalis*, based on published environmental tolerances to these factors. In addition, we used a high-resolution three-dimensional hydrographic model to simulate the probability of spread of *T*. *navalis* larvae within the study area. Climate envelope modeling showed that projected near-future climate change is not likely to change the overall distribution of *T*. *navalis* in the region, but will prolong the breeding season and increase the risk of shipworm establishment at the margins of the current range. Dispersal simulations indicated that the majority of larvae were philopatric, but those that spread over a wider area typically spread to areas unfavourable for their survival. Overall, therefore, we found no substantive evidence for climate-change related shifts in the distribution of *T*. *navalis* in the Baltic Sea, and no evidence for increased risk of spread in the near-future.

## Introduction

There is a worldwide trend towards range expansions in terrestrial and marine systems [1–4], which are being driven – at least in part – by anthropogenically-mediated introductions and global climate change e.g. [5–9]. Poleward range expansions (and in some cases range-contractions poleward from low-latitude limits) have been reported in concert with rising seawater temperature in multiple species including algae [10], plankton [11,12], corals [13], molluscs [8], echinoderms [14], fish [15], and cetaceans [16], although this pattern is not universal (e.g. [17,18]). Within the molluscs, range expansions have been reported for bivalves [12,19], gastropods [18,20–23], and most recently for shipworms [24].

Understanding the factors that determine changes in the distribution of species is crucial for predicting impacts of climate change [25]. Several methods to model these relationships have been developed (reviewed by [26]). One common approach is to identify the environmental factors that correlate with the current geographical range to create an overall “climate envelope” (e.g. [27]). Typically, these models correlate the current geographical distribution (usually presence-absence data) of a species with corresponding distributions of relevant climate variables to infer the species’ environmental requirements. The interpretation of such models is under debate, not least because they identify the current niche – a product of environmental tolerances *plus* dispersal patterns, species interactions, etc. (i.e. the “realised niche”, *sensu* Hutchinson [28]), rather than the potential niche in a different, and perhaps novel, climate/environment (the “idealised niche”, [28]; [29–32]). In contrast, mechanistic models use knowledge of a species’ physiological tolerances to model the idealised niche. These mechanistic models have the benefit of being independent of non-climate factors that can influence the present distribution [30], but may still ignore environmentally-driven plasticity. Regardless of the model type, factors that determine dispersal within the modelled area are rarely included [33], and yet such factors are crucial for the majority of marine organisms whose larvae disperse on ocean currents [34].

Shipworms (Mollusca: Teredinidae) burrow into exposed wood in the oceans, and consequently have few predators and primarily compete for resources with other shipworms [35]. Their distribution has therefore been suggested to be largely determined by physiological tolerances to environmental variables (temperature, salinity, and dissolved oxygen), dispersal by ocean currents (as larvae and adults), and the availability of wood, their primary habitat and food [36]. Thus, they are perhaps ideal subjects for mechanistic climate envelope modeling.

The common shipworm, *Teredo navalis* L., has a global distribution [36] and broad environmental tolerance limits [24,35]. Tolerance ranges vary with life-cycle stage, age, condition of the animal, and exposure time, and adults can survive in a completely closed burrow for at least 6 weeks [37–40]. Range expansions of shipworms have recently been reported on the eastern and western boundaries of the Atlantic [41,42] as well as in the Baltic Sea, [24,43,44]. These expansions can have substantial socioeconomic impacts: shipworm damage to coastal structures costs billions of dollars a year [45,46], and endangers underwater cultural heritage [47].

Dispersal of *Teredo navalis* occurs during the pelagic larval stage, as juveniles/adults in driftwood, and through anthropogenic translocation in the hulls of wooden ships and/or in ballast water [5,48]. Sexually mature *T*. *navalis* release 50,000–2,000,000 feeding planktonic “D-stage” larvae at each spawning event [35], which then feed and grow in the plankton for 17–34 days, depending on food and temperature [49–52]. After this time larvae are competent to settle and metamorphose onto exposed wood, although they can delay settlement for a further 3 weeks [53].

The distribution of *T*. *navalis* in the Baltic Sea system (Baltic, Kattegat, Skagerrak) is limited. This system is characterized by a stable horizontal salinity gradient from marine waters (∼32 PSU) where the Skagerrak meets the North Sea to almost freshwater (∼3 PSU) in the northern Gulf of Bothnia, some 1600 km distant [54]. Circulation in the Baltic is complex with fresh water input at the surface and deeper exchange of North Sea water through the Kattegat [55], which leads to near-permanent stratification of the central Baltic and natural hypoxia in deeper more saline waters. *Teredo navalis* is absent from regions of the Baltic where salinities are ≤ 8 PSU (i.e. the central and northern Baltic). Above 8 PSU, however, *T*. *navalis* is common and is frequently found in the more saline and well-mixed waters of the Kattegat and Skagerrak [56,57].

The aim of this study was to model the likelihood of *Teredo navalis* spreading eastward into the Baltic Sea as a result of projected climate-change during the period 2009–2020. A secondary aim was to identify areas vulnerable to shipworm invasion. We further explored correlations between model predictions and reported shipworm distribution, and the environmental factors that most contribute to changes in that distribution. This was achieved using a biophysical dispersal simulation model together with a mechanistic GIS-based climate envelope model based on known physiological tolerances of *T*. *navalis*.

## Material and Methods

### Climate envelope model

We parameterised our mechanistic model with data on temperature, salinity, and oxygen saturation tolerances for reproduction and larval metamorphosis, which we obtained from the literature (Table 1).

**Table 1:** Climate envelope parameters used for modeling.

Variable	Larval metamorphosis and adult reproduction	Adult reproduction only
Temperature (°C)	≥ 12	≥ 11
Salinity (PSU)	≥ 8	≥ 8
Oxygen (mg O_2_ l^-1^)	≥ 4	≥ 4

Data are lower tolerance limits for metamorphosis of larvae and reproduction of adults of *T*. *navalis* based on literature from Atlantic and Baltic waters (see text for details). Tolerance limits for larval metamorphosis and adult reproduction were used to parameterise the “Surface layer” models, whereas tolerance limits for adult reproduction only were used to parameterise the “Bottom layer” models.

### Temperature

In the Atlantic, *T*. *navalis* is reported to spawn at temperatures ≥ 11°C resulting in a long spawning season, which starts in early summer and lasts until autumn [49,51,52,59]. Correspondingly, larval swimming performance is markedly reduced below 10°C [60] and metamorphosis of larvae is reported to only occur above 12°C [61]. We therefore chose 11° and 12°C as the respective thermal tolerance limits for adult reproduction and larval metamorphosis.

### Salinity

Adult *T*. *navalis* can tolerate a wide range of salinities, although rates of (filter) feeding and wood-boring are reduced at salinities < 7 PSU [42]. Spawning has been reported to occur at 10–35 PSU [62] in the laboratory, which corresponds well with observations of spawning in the Baltic Sea (salinities > 8–10 PSU [43]). On the basis of these data we selected 8 PSU as the functional lower limit for reproduction of adult *T*. *navalis*. Salinities < 5 PSU are lethal to larvae, and larval swimming is strongly reduced below 10 PSU [60]. Metamorphosis of larvae has only been observed at salinities ≥ 8 PSU [43,57], and therefore we chose the latter value as the limiting salinity tolerance for larvae.

### Oxygen

Hypoxic, or anoxic, “dead zones” are increasingly being recognised as an important aspect of marine climate change [63], and the Baltic is no exception to this [64,65]. The effects of changing oxygen tension on *T*. *navalis* are, however, difficult to predict because hypoxia/anoxia tolerance data for *T*. *navalis* are very limited. The few data available indicate that adults can seal their burrows and survive many weeks under anoxic conditions [66], and that oxygen consumption of actively respiring adults varies widely [38,40,67,68]. In the absence of further information we set the minimum oxygen requirement for adult reproduction and larval metamorphosis in *T*. *navalis* to 4 mg O_2_ l^−1^ – a level that has been shown to impede, or limit, these traits in other bivalves [69–72] (Table 1).

### The model

Hydrological data for the bioclimate envelope model were obtained from the Danish Hydrological Institute. Data were obtained from a regional climate model in the MIKE 3 numerical modelling system for 3D flows to produce a nested grid with a spatial resolution of 3 nautical miles in the area Skagerrak – SE Sweden, and 9 nautical miles in the rest of the Baltic Sea. Model output was converted to ASCII files, binned into monthly averages and combined in different algebraic combinations based on Table 1. The output covered two temporal periods: a "hindcast" period (1980–2008) and a "predicted" period (2009–2020) (model data were obtained in 2009/2010; more details are available at [83]). Output for the modelled water body was then binned into two layers: a "Surface layer" comprising the upper 9 m of the water column, and a “Bottom layer” comprising the lowest 2m of the water column (in waters ≤ 9 m deep these two layers overlapped). A climate envelope model for the Surface layer was created by classifying each grid in each time-step as either “favourable” or “unfavourable” for adult reproduction and larval metamorphosis, depending on whether or not modeled values of temperature, salinity and oxygen content exceeded the combined lower bounds of tolerance for those traits (first column, Table 1). An equivalent model for the "Bottom layer" was parameterised using lower tolerance limits only for reproduction of adults (second column, Table 1). The intention was to model the potential for successful reproduction and establishment of shipworm larvae in the “Surface layer” – the primary settlement site for *T*. *navalis* larvae [58], the likelihood that source populations of larvae may exist in deeper waters (“Bottom layer”), and the potential for decadal changes in the degree of coupling between occurrence of favourable conditions in the two layers.

ASCII files were processed in GIS "ModelBuilder" (ESRI ArcMap) to produce maps indicating the frequency of occurrence of favourable conditions in each grid cell over different time periods.

### Model validation

Model results were cross-validated against known shipworm infestation status on wooden panels along the Swedish coast and around the island of Bornholm (Denmark) during the years 2006–2012 (this study), and on groynes along the coast of Germany in 1993–1996 [43].

### Simulation of larval dispersal

Dispersal of *T*. *navalis* larvae was simulated with a biophysical model based on velocity fields from an ocean circulation model, using a particle-tracking routine to generate dispersal trajectories. Velocity fields were modelled with the 3-dimensional ocean circulation model BaltiX [73]. BaltiX is a regional model for the Baltic Sea, Kattegat, Skagerrak and the North Sea (configured from the NEMO ocean engine [74]). BaltiX was applied in hindcast mode for 8 years (1995–2002) with a horizontal resolution of 3.704 km (2 nm), a vertical resolution of 3–22 m, and a temporal baroclinic resolution of 6 min. Dispersal in surface waters (0–12 m) was simulated as particle trajectories lasting for 30 days in May – October calculated with the Lagrangian trajectory model TRACMASS, based on Döös [75]. As there are no available data detailing diurnal or ontogenetic changes in shipworm larval behaviour, larvae were modeled as passive particles. Trajectories were simulated in off-line mode using the velocity fields generated by the BaltiX model with a 3-hour update. Dispersal from selected grid cells to waters with a depth above 100 m in the Baltic Sea, the Danish Straits and the Kattegat was simulated by releasing 98 particles distributed across each grid cell and distributed between 0–12 m depth. This was repeated at 6 time points within each year and repeated for all 8 years resulting in a total of 4704 trajectories per grid cell. Dispersal probabilities from the selected grid cells to receiving grid cells (depth ≤100 m) were calculated as the proportion of trajectories starting at grid cell *j* and ending in grid cell *i*.

**Fig 1:**
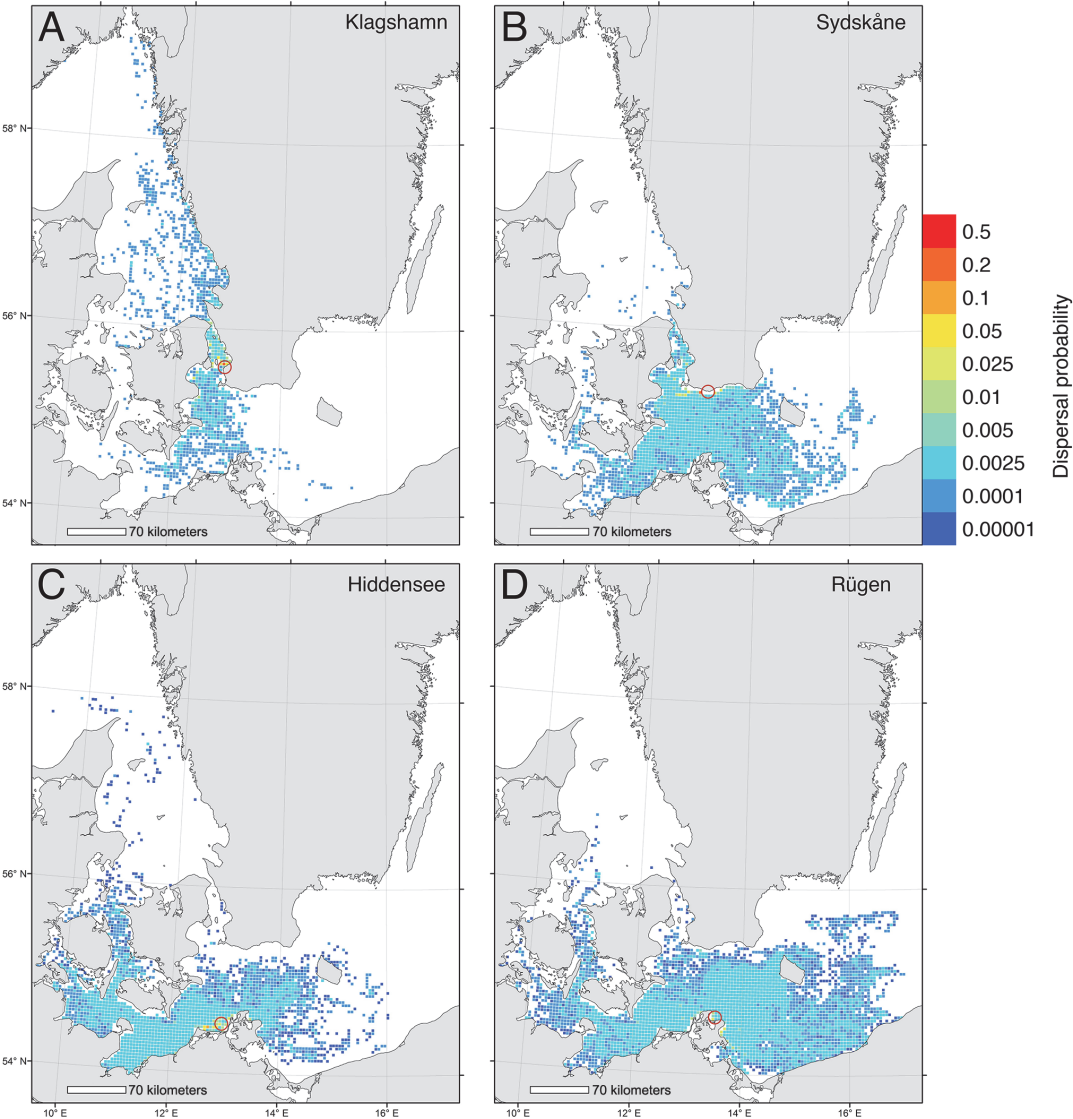
Biophysical simulations showing dispersal of larvae from 4 Baltic locations. Relative larval dispersal probabilities predicted from a biophysical model where larvae were released between May-October and drifted at a depth of 0–12 m for 30 days. Dispersal is shown for 4 seeding locations: (A) Klagshamn, (B) Sydskåne, (C) Hiddensee, (D) Rügen.

**Fig 2:**
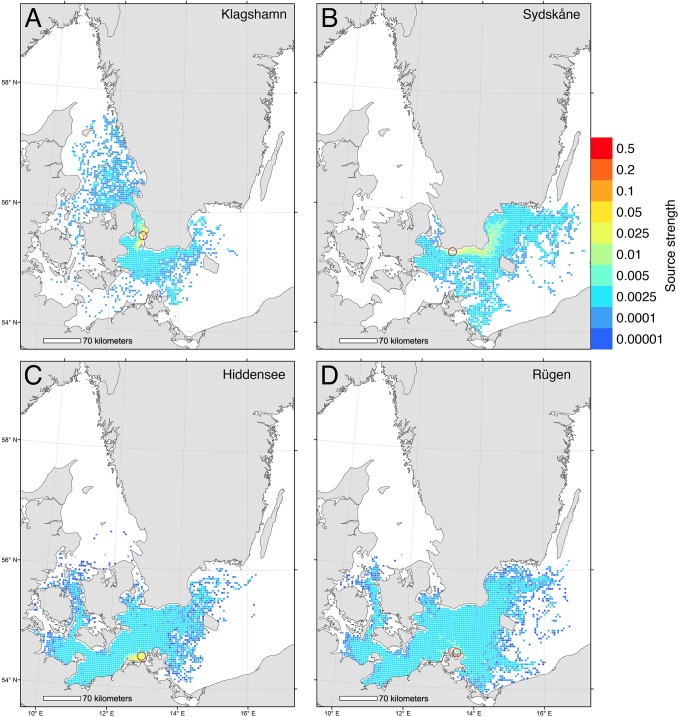
Biophysical simulations showing sources of larvae to 4 Baltic locations. Relative strength of source areas delivering larvae to 4 target areas. Larvae at the source areas were released between May-October and drifted at a depth of 0–12 m for 30 days. Sink areas are shown for 4 locations: (A) Klagshamn, (B) Sydskåne, (C) Hiddensee, (D) Rügen.

From simulated dispersal between all grid cells we calculated the dispersal probability from 4 release areas (Fig. 1). The first area, Klagshamn, is the most southerly site of infestation in Sweden [57], and the second area, Hiddensee, is the most easterly infestation in Germany [43]. In addition we included two release areas further east into the Baltic Sea as reference areas, one on the Swedish coast (Sydskåne) and one on the German coast (Rügen). Finally, we identified the sources of larvae to the selected areas, i.e. the putative spawning sites of simulated larvae that metamorphosed within the target areas (Fig. 2). Dispersal from and to the 4 target areas was plotted by color-coding grid cells using the GIS software ESRI ArcMap.

## Results

### Climate envelope model for reproduction and larval metamorphosis (“Surface layer”)

The occurrence of environmental conditions favourable for reproduction and larval metamorphosis of *T*. *navalis* varied markedly over space and time.

**Fig 3:**
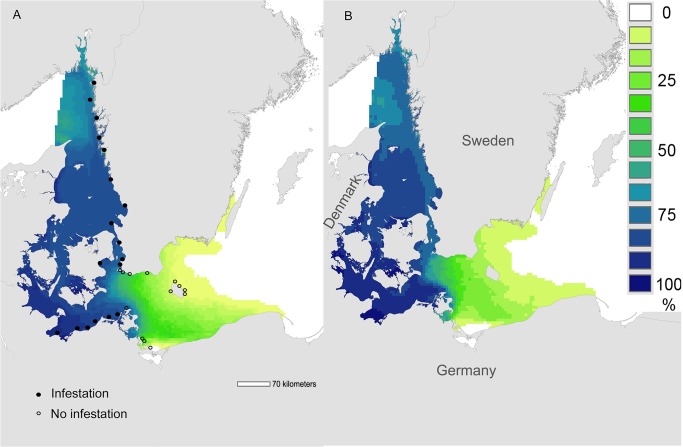
Frequency of occurrence of environmental conditions favourable for adult reproduction and larval metamorphosis in the Surface layer (≤ 9m). Frequency of occurrence (percentage of all months over all years) in which mean salinity, temperature, and oxygen concentrations exceeded tolerance limits for adult reproduction and larval metamorphosis in the upper 9m ("Surface layer") of the water column (Table 1). (A) "Hindcast" period 1980–2008 and (B) "Forecast" period 2009–2020. Filled and empty black circles show infested and non-infested sites, respectively ([24,43,57], this study).

Overall results from the hindcast model (1980–2008) showed that favourable conditions were prevalent (>75% of grid-cell months exceeding tolerance limits, dark blue areas, Fig. 3A) throughout the Skagerrak and the western Baltic, absent in the south-eastern and central Baltic (and in the remainder of the Baltic system, white areas, Fig. 3A), and showed a strong transitional gradient (60–0%) between these two regions (yellow-green areas, Fig. 3A). Patterns for the “forecast” period (2009–2020) were almost identical to those for “hindcast” (*cf* Fig. 3A, 3B), although the smaller sample size of the forecast period (12 years) yielded coarser resolution (Fig. 3B). The absence of favourable conditions in the eastern Baltic was driven by salinity, which was < 8 PSU throughout this region. Salinities in the Skagerrak, Kattegat and western Baltic were routinely > 8 PSU, and here temperature controlled the occurrence of favourable conditions. Oxygen concentration was above the threshold value (4 mg O_2_ l^-1^, Table 1) throughout the modelled space and time, and was never a limiting factor.

**Fig 4:**
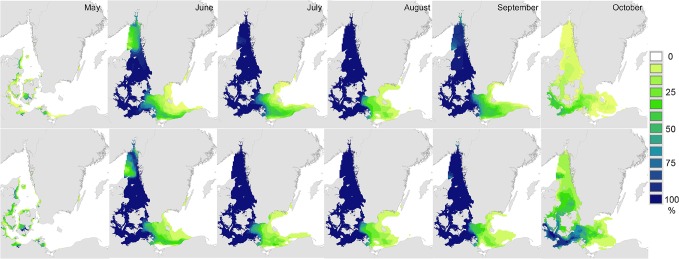
Monthly frequency of occurrence of environmental conditions favourable for adult reproduction and larval metamorphosis in the Surface layer (≤ 9m). Monthly frequency of occurrence (percentage of all years) in which mean salinity, temperature, and oxygen concentrations exceeded tolerance limits for adult reproduction and larval metamorphosis in the upper 9m ("Surface layer") of the water column (Table 1). Upper panels "Hindcast" period 1980–2008; Lower panels "Forecast" period 2009–2020.

The spatial distribution of favourable conditions for reproduction and metamorphosis varied markedly with month of the reproductive period, but were also broadly similar in hindcast and forecast results (Fig. 4). Favourable conditions were first observed in shallow inshore areas of Denmark and Germany in May, spread to the Kattegat and central western Baltic by June, and began to disappear by October. It is notable that the distribution of favourable conditions was slightly more extensive in May of the forecast data (compared to hindcast data, Fig. 4), and that there was a clear extension of favourable conditions later into the season in the western Baltic in the forecast data (Fig. 4).

**Fig 5:**
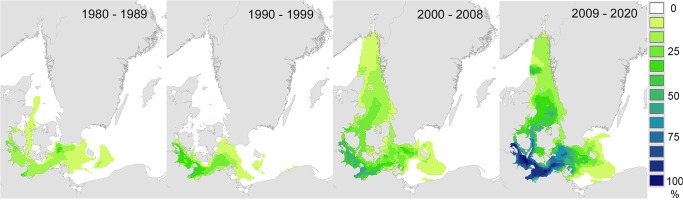
Quasi-decadal patterns of occurrence of environmental conditions favourable for adult reproduction and larval metamorphosis in the Surface layer (≤ 9m) in October. Frequency of occurrence (percentage of years) in which mean salinity, temperature, and oxygen concentrations in October exceeded tolerance limits for adult reproduction and larval metamorphosis in the upper 9m ("Surface layer") of the water column (Table 1).

These data were analysed more closely by summarising model results for October in each of four, approximately decadal, periods (1980–1989, 1990–1999, 2000–2008, 2009–2020). This showed clearly that the prevalence of conditions favourable for reproduction and larval metamorphosis increased over time in the western Baltic, Kattegat and Skagerrak, (Fig. 5). Thus, although the model does not predict extended spatial distribution of *T*. *navalis*, it does predict an extension in the reproductive and larval settlement season of *T*. *navalis* later into the autumn. Investigation of the underlying data showed that this pattern was driven by inter-decadal changes in surface water temperatures, rather than changes in salinity (data not shown).

### Climate envelope model for reproduction only (“Bottom layer”)

**Fig 6:**
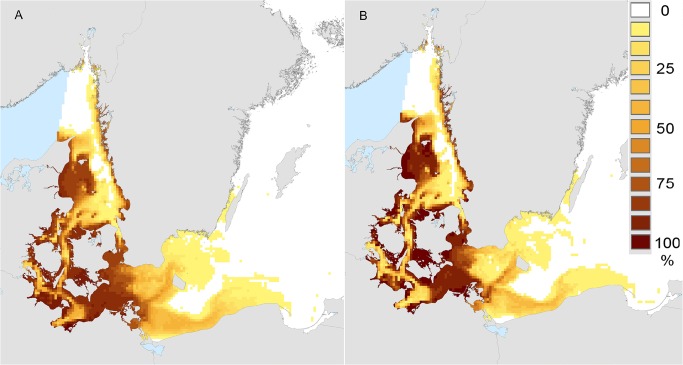
Frequency of occurrence of environmental conditions favourable for adult reproduction (only) in the Bottom layer, (lowest 2m). Frequency of occurrence (percentage of all months over all years) in which mean salinity, temperature, and oxygen concentrations exceeded tolerance limits for adult reproduction and larval metamorphosis in the lowest 2m of the water column ("Bottom layer", Table 1). (A) "Hindcast" period 1980–2008; (B) "Forecast" period 2009–2020.

**Fig 7:**
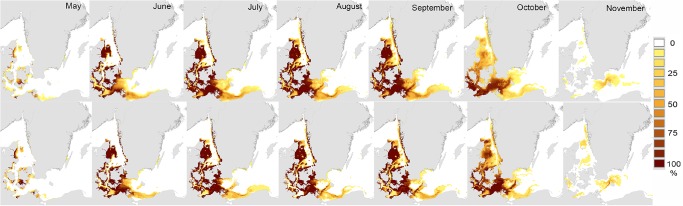
Monthly frequency of occurrence of environmental conditions favourable for adult reproduction (only) in the bottom layer. Monthly frequency of occurrence (percentage of all years) in which mean salinity, temperature, and oxygen concentrations exceeded tolerance limits for adult reproduction and larval metamorphosis in the lowest 2m of the water column ("Bottom layer", Table 1). “Hindcast” period 1980–2008 (above) and “Forecast” period 2009–2020 (below).

Spatiotemporal scenarios (or patterns) of favourable conditions for adult reproduction (only) were broadly similar to those we observed for adult reproduction and larval metamorphosis in the Surface layer (*cf* Figs. 3, 6). In contrast to results for the Surface layer, there was no indication of potentially prolonged reproductive seasons (*cf* Figs. 4, 7). Favourable conditions for reproduction of adults first appeared early in the year (May) in shallow waters (< 25m) and were found until late in the year (November) only in deeper waters (Fig. 7).

### Model validation

Observed shipworm distribution (filled circles, Fig. 3A) corresponded to regions with > 60% occurrence of favourable environmental conditions (Fig. 3A). The most southerly occurrence of *T*. *navalis* in Sweden was at Falsterbo, at the southwestern tip of Sweden. Despite extensive observations over recent years, (2006–2012), no shipworms of any species were observed along the Swedish coast east of this point, or around the island of Bornholm (open circles, Fig. 3A). On the north German coast, the most easterly known location of shipworms is at Hiddensee, Germany [43] – an area where the modelled frequency of favourable environmental conditions was still very high (Fig. 3A).

### Dispersal simulations

The biophysical dispersal model showed that risk of spread of shipworm larvae into the Baltic was relatively low, but nonetheless present. Modeled larval spread from the most easterly known locations of substantial populations of *T*. *navalis* in the Baltic (Klagshamn and Hiddensee, Fig. 3A) showed some spread eastwards, but at low probabilities (< 0.005) and that the majority of larvae dispersed locally, and to the west and north (Fig. 1A,C). We estimated the likelihood that larvae might penetrate even further into the Baltic by modeling spread of larvae from locations eastward of current range limits. Results of these models showed that larvae released on the southern Swedish coast would mostly be swept to the west and south – i.e. out of the Baltic (Fig. 1B), whereas larvae on the north German coast could disperse over a wide area (Fig. 1D).

Model investigations of the sources of larvae recruiting to Klagshamn and Hiddensee showed that recruitment was primarily local, with low probabilities of larvae being transported from distant sites (Fig. 2A,C). Equivalent modeling of the origins of larvae recruiting to areas eastward of current known range limits indicated that, in Sweden, these larvae would originate even further east (Fig. 2B), or across a very wide area (Germany, Fig. 2D).

## Discussion

Our climate envelope model results show clearly that over the last three decades, rising seawater temperatures in the Kattegat and southern Baltic have prolonged the breeding season of *Teredo navalis* into the autumn, and that this is likely to continue at least into the near-future (Fig. 5). Consequently, there is increased risk of greater establishment of *T*. *navalis* at the eastern margins of the current distribution. This may lead to small extensions in the current range. Our climate envelope model results did not support previous suggestions that *T*. *navalis* is expanding into the Baltic [24,43], or that it is likely to do so as a result of near-future climate change – indeed we found no substantive evidence for future range-expansions in the coming decade (Figs. 3, 6). These latter results are reinforced by results from our dispersal simulations (Figs. 1, 2), which indicated limited opportunities for further spread into the Baltic – especially along the Swedish coast.

Our finding that over the last 30 years, the duration of the breeding season has extended later into the year (Fig. 5) is reflected in independent observations from field surveys of *T*. *navalis* in the area. In the 1970’s adult *T*. *navalis* in the Skagerrak/Kattegat region brooded larvae from June to August [76] and larvae were observed to settle and recruit between July and September [61]. Some thirty years later, adult brooding was observed both earlier and later in the season (May to September, [43]), and larval recruitment was observed to last until the end of October (in 2004–2006). These field observations provide important confirmatory support for the results from our model, and provide supportive evidence that mechanistic climate envelope models can provide valuable projections even when based on relatively few biological parameters (Table 1) [30].

Further support for our model comes from the observation that range-margins of *T*. *navalis* in the Baltic corresponded very closely with the modeled limits of favourable conditions for reproduction in surface layers (Fig. 3A). Today, established shipworm populations are only found in areas where the frequency of occurrence of favourable conditions is > 60% (i.e. the majority of the summer months, and years, during which reproduction can take place, Fig. 3A, Fig. 4). The absence of known shipworm populations in areas where favourable conditions are less frequent (green-yellow, Fig. 3A) may indicate that temperature / salinity conditions in those regions are favourable for too short a period to permit successful development and recruitment of larval shipworms, and/or that these conditions exist in only some years. Under the latter scenario, periodic outbreaks of *T*. *navalis* may occur in years with higher temperatures or salinities – both of which favour reproduction (Table 1).

Simulations of potential for spread of *T*. *navalis* at the range-margins indicated that the great majority of larvae released from Klagshamn (Sweden) and Hiddensee (Germany) were retained close to these areas in the western Baltic (Fig. 1A,C), and that larvae recruiting to these populations tended to have local origins (Fig. 2A,C). Interestingly, larvae released to the east of current range margins had low probabilities of spread further east (Fig. 1B,D). Similarly, larvae recruiting to these more easterly locations had moderate probabilities of originating from farther east (Fig. 2B,D). These results suggest that even if *T*. *navalis* populations were able to spread eastwards into southern Sweden, their larvae would be likely to be swept westwards during their dispersal. Genetic analyses of shipworm populations would be a valuable addition to our findings, and help greatly in clarifying the actual patterns of dispersal and population structures in this region.

On larger scales we found no evidence for substantive changes in the distribution of shipworms over time. With the exception of the prolonged breeding season in surface waters mentioned above (Fig. 5), spatiotemporal patterns of conditions favourable for shipworm reproduction and metamorphosis were similar across all periods (hindcast and forecast) and water layers (Surface and Bottom, Figs. 3A,B and 6A,B). This finding contrasts with a recent claim that shipworms are indeed spreading into the Baltic Sea [24]. Borges *et al*.’s claim is based on observations of shipworm distribution in the region and the supposition that *T*. *navalis* may be able to adapt to salinities even lower than its current tolerance limit ([24] p. 7). Regarding distributions, records from Zingst (Germany) almost a century ago testify to shipworm activity very close to the present range margin (Becker 1938, in [77]), and German observations from the 1990’s [43] and 2012 (Rostock University, pers. comm.) show variability but no strong evidence for range shifts. Similarly, along the Swedish coast there is also no indication of range expansion: the southern margin of *T*. *navalis’* distribution at Klagshamn has not changed for the last thirty years (*cf*. [57] Fig. 3).

The suggestion that *T*. *navalis* may be able to adapt to lower salinities is interesting. That environmental tolerances do not change over time is an inherent assumption of correlative climate envelope models (used by Borges *et al*. [24]), and is typically also assumed (though not required) in mechanistic models (used here). Incorporating adaptive capacity into mechanistic climate envelope models is an exciting possibility [31], but doing this here would require knowledge of the additive genetic variance for salinity tolerance in *T*. *navalis* populations in the south-western Baltic – data that are currently lacking. Consequently any conclusions regarding the likelihood that *T*. *navalis* will adapt to salinity changes in this region must remain speculative.

Several studies have highlighted the capacity for mechanistic climate envelope models to provide robust predictions of species distributions, especially in novel climates arising from climate change [30,31]. Climate simulations for the Baltic Sea [78] project increased precipitation and reduced salinity in the coming decades. As our modeling showed that salinity – rather than temperature – tolerances determined the spatial limits for reproduction and metamorphosis in the southern Baltic, we suggest that there is very low risk of range-expansion of *T*. *navalis* into the Baltic Sea during the coming decades.

Logistic constraints limited our ability to test our model for sensitivity to the chosen tolerance parameters (Table 1). Other authors have suggested that establishment of *T*. *navalis* may be limited by salinities different from the values used here (9 PSU [79]; 7 PSU [24]; *vs* 8 PSU, Table 1). As noted earlier, definitive data on salinity, temperature, and (especially) oxygen tolerances of *T*. *navalis* larvae from the southwestern Baltic are lacking (*cf* [60] for tolerances for US populations of *T*. *navalis*). Nonetheless our choice of threshold values for modeling is at least partly corroborated by the strong correspondence between observed distributions of *T*. *navalis* and the modelled occurrence of favourable conditions for their reproduction and larval metamorphosis (Fig. 3A).

Large numbers of archaeologically important wooden shipwrecks in the southern and eastern Baltic provide abundant, potentially suitable, habitat for wood boring pests [47]. To date the risk that such wrecks may be attacked by shipworms has been mitigated by methods such as *in situ* protection of wooden wrecks [80] – a method that not only preserves the historical remains, but also might reduce further spread of shipworms. Other mitigation options that have been discussed include removing ‘stepping stone’ nodes in shipworm connectivity matrices by, for example, covering wooden structures with plastic. Such an approach might be interesting to test on the wooden groynes around Hiddensee, where our biophysical model indicated high local recruitment (Fig. 1C). A better understanding of the impacts of larval behaviour (which is poorly understood) and passive dispersal of adults in driftwood are, however, required before comprehensive connectivity models can be constructed and tested. In this context the role of larval behaviour in dispersal [81,82], as well as more accurate, and population-specific, measures of salinity and temperature tolerances (Table 1) are required in order to refine the models we present here.

In summary, we found that current climate change is causing an extension in the breeding period of *Teredo navalis* in the western and southern Baltic, but that this has not resulted in a range expansion of this species over the past 30 years, and is not likely to do so in the near-future. Scenarios for the coming century suggest that freshening of the Baltic Sea will further constrain the distribution of *T*. *navalis*, lessening the threat to the considerable underwater architectural heritage of the region.
